# Proteomic analysis of cerebrospinal fluid extracellular vesicles reveals synaptic injury, inflammation, and stress response markers in HIV patients with cognitive impairment

**DOI:** 10.1186/s12974-019-1617-y

**Published:** 2019-12-05

**Authors:** Debjani Guha, David R. Lorenz, Vikas Misra, Sukrutha Chettimada, Susan Morgello, Dana Gabuzda

**Affiliations:** 1 Department of Cancer Immunology and Virology, Dana-Farber Cancer Institute, CLS 1010, 450 Brookline Ave, Boston, MA 02215 USA; 2 Departments of Neurology, Neuroscience and Pathology, Mount Sinai Medical Center, New York, NY USA; 3 Department of Neurology, Harvard Medical School, Boston, MA USA

**Keywords:** HIV, Cognitive impairment, CSF, Extracellular vesicles, Proteomics

## Abstract

**Background:**

Extracellular vesicles (EVs) are nano-sized particles present in most body fluids including cerebrospinal fluid (CSF). Little is known about CSF EV proteins in HIV+ individuals. Here, we characterize the CSF EV proteome in HIV+ subjects and its relationship to neuroinflammation, stress responses, and HIV-associated neurocognitive disorders (HAND).

**Methods:**

CSF EVs isolated from 20 HIV+ subjects with (*n* = 10) or without (*n* = 10) cognitive impairment were characterized by electron microscopy, nanoparticle tracking analysis, immunoblotting, and untargeted LC/MS/MS mass spectrometry. Functional annotation was performed by gene ontology (GO) mapping and expression annotation using Biobase Transfac and PANTHER software. Cultured astrocytic U87 cells were treated with hydrogen peroxide for 4 h to induce oxidative stress and EVs isolated by ultracentrifugation. Selected markers of astrocytes (GFAP, GLUL), inflammation (CRP), and stress responses (PRDX2, PARK7, HSP70) were evaluated in EVs released by U87 cells following induction of oxidative stress and in CSF EVs from HIV+ patients by immunoblotting.

**Results:**

Mass spectrometry identified 2727 and 1626 proteins in EV fractions and EV-depleted CSF samples, respectively. CSF EV fractions were enriched with exosomal markers including Alix, syntenin, tetraspanins, and heat-shock proteins and a subset of neuronal, astrocyte, oligodendrocyte, and choroid plexus markers, in comparison to EV-depleted CSF. Proteins related to synapses, immune/inflammatory responses, stress responses, metabolic processes, mitochondrial functions, and blood-brain barrier were also identified in CSF EV fractions by GO mapping. HAND subjects had higher abundance of CSF EVs and proteins mapping to GO terms for synapses, glial cells, inflammation, and stress responses compared to those without HAND. GFAP, GLUL, CRP, PRDX2, PARK7, and HSP70 were confirmed by immunoblotting of CSF EVs from subjects with HAND and were also detected in EVs released by U87 cells under oxidative stress.

**Conclusions:**

These findings suggest that CSF EVs derived from neurons, glial cells, and choroid plexus carry synaptic, immune/inflammation-related, and stress response proteins in HIV+ individuals with cognitive impairment, representing a valuable source for biomarker discovery.

## Introduction

While the introduction of combination antiretroviral therapy (ART) has dramatically improved overall health and life expectancy of HIV-positive (HIV+) individuals, HIV-associated neurocognitive disorders (HAND) remain a major cause of morbidity. HAND, consisting of asymptomatic neurocognitive impairment (ANI), mild neurocognitive disorder (MND), and HIV-associated dementia (HAD), affects up to 20–50% of HIV-infected individuals in the era of combination ART [1–3]. The incidence of HAD has decreased on current ART regimens, but the prevalence of milder forms of HAND has increased [1, 3]. Although clinical significance of ANI remains unclear, HIV patients diagnosed with ANI have elevated risk of progressing to MND or HAD [4]. The progression and severity of HAND are highly variable, and little is known about the underlying mechanisms. Persistent inflammation, oxidative stress, metabolic disturbances, blood-brain barrier (BBB) dysfunction, and ART drug neurotoxicity are likely to be contributing factors [1, 5–10]. Discovering prognostic and diagnostic biomarkers is important to identify the onset and progression of HAND in ART-treated HIV+ individuals and to inform approaches to new therapeutics.

Cerebrospinal fluid (CSF) is in part derived from brain parenchyma and serves as an important indicator of neurological diseases. Elevated CSF neurofilament light chain (NFL), neopterin, soluble CD14 (sCD14), inflammatory cytokines, and chemokines are biomarkers frequently associated with HAND in HIV patients, particularly those with non-suppressed plasma viremia [11–14]. However, HAND-specific biomarkers are still lacking [15]. Proteomic analyses have identified changes in the CSF proteome across the spectrum of neurocognitive disorders in HIV patients [16–20], suggesting this approach may lead to discovery of HAND biomarkers, along with better understanding of underlying pathophysiology.

Extracellular vesicles (EVs) have been identified as biomarkers for neurological diseases including Alzheimer’s disease [21, 22], Parkinson’s disease [23], and multiple sclerosis [24, 25]. EVs are generated from most cell types and released into body fluids, including CSF. Depending on size and cellular origin, EVs are classified as exosomes (50–150 nm, originating from endosomal multivesicular bodies) or microvesicles (200 nm–1 μm, originating from plasma membranes). EVs carry proteins, lipids, and nucleic acids from parental cells and transfer cargo to recipient cells to mediate physiological and pathological functions [26, 27]. In neurodegenerative disorders, EVs produced by central nervous system (CNS) cells carry and deliver abnormal aggregated proteins [28–30], which may favor amplification and spread of protein misfolding diseases [31] in addition to providing a concentrated source of relevant biomarkers. Proteomic analyses of CSF EV proteins could lead to discovery of new predictive biomarkers that are otherwise difficult to detect in CSF due to their low abundance. However, despite potential importance of EVs in the pathogenesis of neurological diseases, CSF EV proteins in these diseases remain poorly characterized.

EVs are proposed to play important roles in HIV pathogenesis [32–34]. HIV infection induces release of EVs from various cell types including immune and brain cells [35–37], and EVs released from HIV-infected cells transport viral and host proteins that can facilitate viral dissemination through body fluids [38–42]. Previous studies have shown that plasma EVs have elevated abundance and size in HIV-infected individuals [34, 43] and carry viral proteins [44], pro- and anti-inflammatory cytokines/chemokines [41, 42], and markers related to immune activation, oxidative stress, and cognitive impairment [34, 45]. In comparison to peripheral blood EVs, little is known about the cellular origin, cargo, and functional roles of CSF EVs in the context of HIV infection and HAND. CSF EV cargo in normal subjects [46–48], multiple sclerosis [24, 25], and traumatic brain injury [49] has been characterized in limited studies. We previously reported that increased CSF EVs in ART-treated HIV+ subjects correlate with the neuronal injury biomarker CSF NFL [50], suggesting a potential role of CSF EVs in HIV-associated neurocognitive impairment. Here, we characterized the CSF EV proteome in HIV-infected subjects and its relationship to neurocognitive impairment by untargeted mass spectrometry.

## Methods

### Characterization of biological samples

CSF samples from 20 HIV+ subjects were collected between 1998 and 2013 by the National NeuroAIDS Tissue Consortium (NNTC) (Manhattan HIV Brain Bank, National Neurological AIDS Bank, California NeuroAIDS Tissue Network, and Texas NeuroAIDS Research Center) and stored at − 80 °C. All subjects were enrolled with written informed consent and institutional review board (IRB) approval at each study site. HAND clinical diagnoses were determined using established criteria [51] based on formal neurocognitive testing and neurological evaluation. Inclusion criteria were use of combination ART, age > 40 years, and undetectable CSF viral load (VL) (< 50 HIV RNA copies/ml). We also included one subject with detectable CSF VL (328 copies/ml).

### Cell culture and treatment

Human glioblastoma U87 cells were cultured in Dulbecco’s modified Eagle’s medium (DMEM) supplemented with 10% exosome-free fetal bovine serum (FBS), 100 U/ml penicillin, and 100 mg/ml streptomycin. The cells were maintained at 37 °C in a humidified atmosphere of 5% CO_2_. To induce oxidative stress, U87 cells were treated with increasing concentrations (10, 25, 50, 100, 250, 500, 1000 μM) H_2_O_2_ for 4 h in serum-free medium_._ After 4 h treatment, cells were washed with phosphate-buffered saline (PBS) and cultured in exosome-depleted medium for 72 h. Control and H_2_O_2_-treated U87 cells and culture supernatants were harvested for immunoblotting and EV isolation.

### EV isolation from CSF and U87 cell culture supernatant

CSF samples (400 μl) were centrifuged at 3000*×g* for 15 min to remove cellular debris. Supernatants were immunoglobulin (Ig) depleted by incubation with protein A/G agarose beads and protein-L beads at room temperature for 1 h. Twelve common abundant proteins (α1-Acid glycoprotein, fibrinogen, α1-antitrypsin, haptoglobin, α2-macroglubulin, IgA, IgG, IgM, albumin, apolipoprotein A-I, apolipoprotein A-II, and transferrin) were depleted using Proteome Purify-12 immunodepletion resin (R & D Systems) at room temperature for 30 min. Abundant protein-depleted CSF samples were then incubated overnight at 4 °C with ExoQuick exosome precipitating reagent (System Biosciences, Inc., Mountain View, CA). The mixture was centrifuged at 1500*×g* for 30 min and EV pellets were resuspended in 20 μl PBS. EV-depleted CSF was concentrated 5-fold by passing through a 10K Amicon filter and stored at − 80 °C until further processing. EVs from control and H_2_O_2_-treated U87 cells were isolated from 150 ml cell culture media by ultracentrifugation. In brief, cell culture media was centrifuged at 300*×g* at 4 °C for 10 min to remove floating cells. Supernatants were passed through a 0.2 μm filter to remove contaminating apoptotic bodies, larger microvesicles, and residual cell debris. The flow through was centrifuged at 150,000*×g* at 4 °C for 90 min to pellet exosomes and smaller vesicles. The supernatant was removed and EV pellet was resuspended in 35 ml cold PBS and then centrifuged at 150,000*×g* for 90 min. The resulting EV pellet was resuspended in 100 μl PBS.

### Nanoparticle tracking analysis (NTA) and transmission electron microscopy (TEM)

CSF and U87 EV concentrations and sizes were measured by nanoparticle tracking analysis (NTA) on a ZetaView instrument (Particle Metrix, Germany). For electron microscopy, CSF EVs isolated from 300 μl CSF were suspended in 10 μl PBS containing 1% paraformaldehyde. EV samples were adsorbed for 1 min to a formvar/carbon coated grid and fixed for 5 min in 1% glutaraldehyde. EVs were washed on a drop of water and stained with 1% uranyl acetate for 30 s. EV morphology was analyzed with a Tecnai G2 Spirit BioTWIN transmission electron microscope (TEM) equipped with an AMT 2 k CCD camera at the Harvard University TEM core.

### SDS PAGE and Immunoblotting

CSF EV and corresponding EV-depleted CSF samples were mixed with an equal volume of radioimmunoprecipitation assay (RIPA) lysis buffer (Triton X-100 1%, NaCl 150 mM, sodium deoxycholate 0.5%, Tris-HCL 50 mM, SDS 0.1%, pH 7.4). U87 cells and EVs were also lysed with RIPA buffer and protein concentration was estimated by BCA assay. Modified Laemmli 4X sample buffer was added to lysed EVs and EV-depleted CSF samples and boiled for 5 min. Equal volumes of CSF EVs and corresponding EV-depleted CSF samples were loaded on 4–12% gradient polyacrylamide gels. Forty micrograms of U87 cell and EV lysates were loaded in each lane. After electrophoresis, protein bands were transferred onto PVDF membranes for 1.5 h at room temperature. The membranes were blocked with 4% non-fat milk for 1 h and probed at 4 °C for human IgG (Sigma-Aldrich; Merck) CD9 (Santa Cruz Biotechnology), CD81 (System Biosciences), heat-shock protein 70 (HSP70) (System Biosciences), flotillin-1 (FLOT-1) (BD Biosciences), glial fibrillary acidic protein (GFAP) (Abcam), glutamine synthase (GLUL) (Abcam), parkinsonism associated deglycase (PARK7) (Abcam), and C-reactive protein (CRP) (Abcam). After secondary antibody treatment, blots were developed with enhanced chemiluminescence (ECL). Images were captured using the Bio-Rad ChemiDoc™ Imaging System. Densitometric quantification was carried out with ImageJ software.

### Mass spectrometry and protein sequence analysis

To isolate CSF EVs for proteomics, CSF samples were pre-cleared using Protein A/G PLUS-Agarose beads (Santa Cruz Biotechnology) and Proteome Purify™ -12 kit (R&D systems). Immunodepleted CSF samples were characterized by SDS-PAGE and silver staining before EV isolation (Additional file 6: Figure S1a). CSF EVs were precipitated using ExoQuick reagent, digested in 0.5% RapiGest, and boiled at 100 °C for 5 min. Protein bands in EV fractions and EV-depleted CSF samples were separated on polyacrylamide gels and visualized by silver staining (Pierce) to estimate protein content. IgG depletion was confirmed by immunoblotting (Additional file 6: Figure S1b). EV and EV-depleted CSF proteins from corresponding CSF samples (400 μl) were analyzed by the Taplin Biological Mass Core Facility using an ABSciex 4800Plus MALDI-TOF/TOF mass spectrometer. In brief, proteins were reduced using 1 mM DTT (in 50 mM ammonium bicarbonate) for 30 min at 60 °C. Samples were then cooled to room temperature and iodoacetamide (stock in 50 mM ammonium bicarbonate) was added to a concentration of 5 mM for 15 min in the dark at room temperature. DTT was then added to a 5 mM concentration to quench the reaction. EV and EV-depleted CSF proteins were digested overnight using 5 ng/μl sequence grade trypsin (Promega, Madison, WI) at 37 °C. Samples were then desalted by an in-house desalting column. Peptides were extracted by removing the ammonium bicarbonate solution, followed by one wash with a solution containing 50% acetonitrile and 1% formic acid. Extracts were then dried in a speed-vac (~ 1 h) and stored at 4 °C until analysis. On the day of analysis, samples were reconstituted in 5–10 μl of HPLC solvent A (2.5% acetonitrile, 0.1% formic acid). A nano-scale reverse-phase HPLC capillary column was created by packing 2.6 μm C18 spherical silica beads into a fused silica capillary (100 μm inner diameter × ~ 30 cm length) with a flame-drawn tip [52]. After equilibrating the column, each sample was loaded via a Famos auto sampler (LC Packings, San Francisco CA). A gradient was formed, and peptides eluted with increasing concentrations of solvent B (97.5% acetonitrile, 0.1% formic acid). As peptides eluted, they were subjected to electrospray ionization and then entered into an LTQ Orbitrap Velos Pro ion-trap mass spectrometer (Thermo Fisher Scientific, Waltham, MA). Peptides were detected, isolated, and fragmented to produce a tandem mass spectrum of specific fragment ions for each peptide. Peptide sequences (and hence protein identity) were determined by matching protein databases with the acquired fragmentation pattern using Sequest software (Thermo Fisher Scientific, Waltham, MA) [53]. All databases included a reversed version of all sequences, and the data was filtered to 1–2% peptide false discovery rate (FDR). Common high abundance proteins were excluded from downstream analysis because the majority are likely to be blood-derived (Additional file 1).

### MTT assay

U87 cells were seeded in 96-well plates at a density of 5 × 10^4^ cells/well. Cells were treated in triplicate with 10, 25, 50, 100, 250, 500, or 1000 μM H_2_O_2_ for 4 h. The media was removed, cells were washed with PBS and cultured in fresh DMEM supplemented with 10% FBS, 100 U/ml penicillin, 100 μg/ml streptomycin, and 0.5 mg/ml MTT reagent (Sigma-Aldrich; Merck) for 4 h in the dark at 37 °C. The resultant blue formazan crystals in cells were dissolved in dimethyl sulfoxide. Optical density in each well was determined at 570 nm using a microplate reader.

### Detection of reactive oxygen species (ROS)

Intracellular ROS was measured using dichlorodihydrofluorescein diacetate (H_2_DCFDA) reagent. Cells were treated with 50, 100, or 250 μM H_2_O_2_ for 4 h. Mock and H_2_O_2_-treated cells were loaded with 20 μM H_2_DCFDA for 45 min at 37 °C. Following two washes with pre-warmed PBS, fluorescence was measured at 485ex/535em nm or analyzed with a fluorescence microscope. Data were normalized to corresponding protein concentrations.

### Data processing and statistical analysis

Functional annotation was performed by gene ontology (GO) mapping and expression annotation using Biobase (genexplain.com/transfac) and PANTHER (pantherdb.org). Proteins identified by ≥ 2 peptide count were included for analyses. Heatmaps were generated using log_2_ transformed peptide intensities from LC-MS analyses using R (version 3.5.1). Immunoblot bands were quantitated using ImageJ software and graphical representations plotted in GraphPad prism version 7.0. Densitometric values represent mean + SEM. Differences in CSF EV concentrations and number of proteins detected in CSF EV between groups were analyzed by Mann-Whitney *U* test. Differences between HAND and control subjects, or between control and H_2_O_2_-treated U87 cell lysates and EVs, were analyzed by one-way ANOVA. Differences in ROS induction and cell viability between control and H_2_O_2_-treated U87 cells were analyzed by *t* test.

## Results

### Study cohort

Demographic and clinical characteristics of the study cohort are summarized in Table 1. The cohort consisted of 20 HIV+ subjects with advanced disease (90% with nadir CD4 count < 200 cells/μl and median nadir CD4 count 31 cells/μl) and therefore at high risk of CNS injury. Subjects were male, 75% white, with median age 52.2 years (interquartile range [IQR], 46.9–58.7 years). Median duration of HIV infection was 13.3 years (IQR, 10–21). Ten HIV+ subjects were cognitively impaired (9 had HAND diagnoses, of which 2, 3, and 4 subjects had ANI, MND, and HAD, respectively, while 1 had neuropsychological impairment due to other causes [NPI-O]). HAD subjects were included to evaluate changes in the CSF EV proteome in severe as well as milder forms of HAND. The majority (90%) were on ART (80% on protease inhibitors), with 65% and 94% having undetectable plasma and CSF VL, respectively. Subjects with HAND were mostly viremic (60%) and had low median CD4 count of 157 cells/μl (IQR, 75–182), while those without HAND were mostly aviremic (90%) and had a higher median CD4 count of 262 cells/μl (IQR, 146–411). With regard to substance use and comorbidities, the prevalence of smoking, alcohol use, HCV seropositivity, and depression was 75%, 15%, 45%, and 44% among all HIV+ subjects, respectively, with no significant differences by HAND status, while cocaine use and cerebrovascular disease were more frequent in HAND subjects (40% and 30%, respectively). Seven HAND and 3 non-HAND subjects died within 6 years following the sample date; causes of death included cirrhosis, end stage renal disease, pneumonia, lymphoma, and non-AIDS-defining cancers including metastatic adenocarcinoma, metastatic anal squamous cell carcinoma, lung cancer, and renal cell carcinoma. Only one of these 10 autopsied cases had HIV encephalitis.

**Table 1:** Demographic and clinical characteristics of the study cohort

	HIV+ (*n* = 20)	no HAND (*n* = 10)	HAND (*n* = 10)
Age (years)	52.2 (47–59)	51.3 (46.-61)	52.3 (50–58)
Gender (male, *n*, %)	20 (100)	10 (100)	10 (100)
Race (*n*, %)			
Black	5 (25)	2 (20)	3 (30)
White	15 (75)	8 (80)	7 (70)
Smoking (*n*, %)	12 (75)	7 (70)	5 (83)
Alcohol use (*n*, %)	3 (15)	1 (10)	2 (20)
Cocaine use (*n*, %)	4 (20)	0 (0)	4 (40)
Hepatitis C seropositivity (*n*, %)	9 (45)	5 (50)	4 (40)
Cerebrovascular disease (*n*, %)	3 (15)	0 (0)	3 (30)
Depression (*n*, %)	8 (44)	5 (50)	3 (37)
Duration of HIV infection (years)	13.3 (10–21)	15.5 (13–21)	11.5 (6–19)
HIV RNA			
Plasma VL	700 (48–44,131)	38.4 (26–17,774)	4064 (700–236,292)
Plasma (< 50 copies/ml)	13 (65)	9 (90)	4 (40)
CSF (< 50 copies/ml) *	16 (94)	9 (100)	7 (87)
CD4 count (cells/μl)	162 (116–373)	262.5 (146–411)	157 (75–182)
< 350 cells/μl	13 (65)	5 (50)	8 (80)
Nadir CD4 count (cells/μl)	31.5 (12–64)	15 (12–46)	55 (23–71)
< 200 cells/μl (*n*, %)	18 (90)	9 (90)	9 (90)
CSF WBC (cells/μl)	1 (0–2)	2 (1–2)	0 (0–1)
ART use (*n*, %)	18 (90)	10 (100)	8 (80)
Protease inhibitors	16 (80)	8 (80)	8 (80)
Nucleoside RT inhibitors	17 (85)	10 (100)	7 (70)
Integrase inhibitors	4 (20)	2 (20)	2 (20)
HIV encephalitis	1 (5)	0 (0)	1 (10)**

*Abbreviations*: *HAND* HIV-associated neurocognitive disorders, *IQR* interquartile range, *VL* viral load, *WBC* white blood cells, *ART* antiretroviral therapy, *RT* reverse transcriptase, Data represent median (IQR) unless otherwise indicated; *Not available for 3 subjects. ** HIV encephalitis was diagnosed at autopsy in 1 subject with MND. Among subjects with HAND, 7 died within 6 years following the sample date; causes of death included cirrhosis, renal disease, pneumonia, lymphoma, metastatic adenocarcinoma, metastatic anal squamous cell carcinoma, lung cancer, and renal cell carcinoma

### Characterization of CSF EVs

**Fig. 1:**
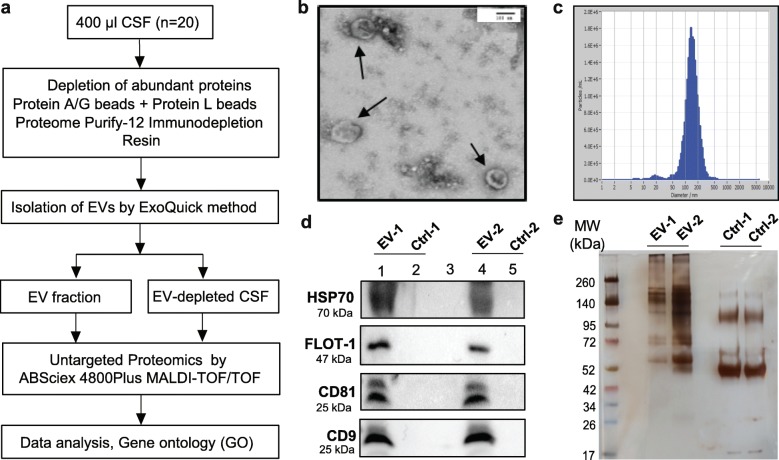
Characterization of CSF EVs from HIV+ subjects. **a** Workflow for proteomic analyses of CSF EVs and EV-depleted CSF. **b** Transmission electron micrograph of whole-mounted CSF EVs from a representative HIV+ subject. EVs are indicated with black arrows. Scale bar = 100 nm. **c** Histogram of CSF EV size distribution by nanoparticle tracking analysis (NTA) from a representative HIV+ subject. **d** Immunoblotting for exosome markers Hsp70, FLOT-1, CD81, and CD9 in CSF EV fractions from two representative HIV+ subjects. EV-depleted CSF from the corresponding samples were used as controls (Ctrl). **e** Silver staining of EV and EV-depleted CSF proteins (Ctrl) from two representative HIV+ subjects. Proteins were separated by SDS-PAGE prior to staining. MW denotes molecular weight markers

CSF EVs were isolated and evaluated for morphological and molecular characteristics. The workflow is shown in Fig. 1a. TEM revealed both round and cup-shaped vesicles with diameter between 50 and 150 nm, corresponding to size of exosomes (Fig. 1b). The average diameter of CSF EVs was 126 nm with a peak at 100–150 nm (Fig. 1c). However, some smaller (< 50 nm) as well as larger vesicles (> 150 nm) were also detected; hence, particles were termed EVs rather than exosomes. EV concentrations ranged between 10^9^ and 10^12^ particles/ml with a mean value of 1.9 × 10^11^ particles/ml. Isolated EVs were further characterized by immunoblotting for exosome markers CD9, CD81, FLOT-1, and HSP70. Concentrated EV-depleted CSF from the corresponding samples were used as controls. CD9, CD81, FLOT-1, and HSP70 were enriched in EV fractions compared to EV-depleted CSF samples (Fig 1d). CSF EV proteins were characterized by silver staining, which indicated depletion of abundant proteins and distinct patterns of protein bands in EV fractions compared to EV-depleted CSF (Fig. 1e) (Additional file 6: Figure S1). The endoplasmic reticulum (ER) membrane markers calnexin and ERP72 were not detected in CSF EV fractions by western blotting, suggesting EV preparations were free of ER contamination (Additional file 6: Figure S2).

### Proteomic analysis of CSF EVs and EV-depleted CSF

**Fig. 2:**
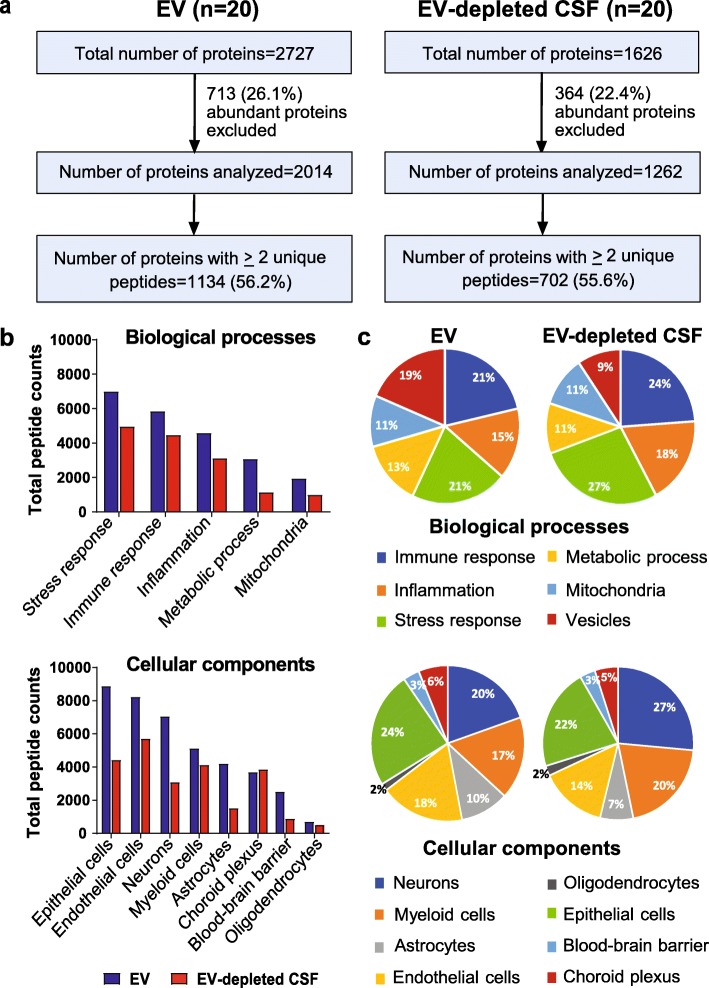
Comparison of CSF EV and EV-depleted CSF proteins in HIV+ subjects (*n* = 20). **a** Flowcharts summarizing numbers of total and abundant proteins detected in CSF EV fractions and EV-depleted CSF by untargeted proteomics. **b** Comparison of total peptide counts for proteins mapped to selected biological process and cellular component ontology terms in EV fractions and EV-depleted CSF. **c** Pie-charts illustrating the percentage of proteins mapped to different biological process and cellular component categories in EV fractions and EV-depleted CSF by GO analysis and expression annotation

To identify protein cargo in CSF EVs from HIV+ subjects, isolated EV fractions and corresponding EV-depleted CSF samples were subjected to untargeted LC/MS/MS analysis. Combined analyses from 20 HIV+ subjects identified a total of 2727 and 1626 proteins in EV and EV-depleted CSF samples, respectively, using the SEQUEST search algorithm with a 1% FDR threshold (Fig. 2a). Proteins detected in EV and EV-depleted CSF samples are shown in Additional files 2 and 3, respectively. Unique peptides per protein in EV fractions ranged from 1 to 172 (median 2) and in EV-depleted CSF from 1 to 50 (median 2), with 12.4% and 12.2% average sequence coverage, respectively. Previous studies have shown that albumin, antitrypsin, apolipoproteins, cystatin, haptoglobin, immunoglobulins, macroglobulins, prostaglandins, and transferrin are highly abundant in CSF irrespective of disease status [17, 18, 54–57]. In EV fractions and EV-depleted CSF, 26% and 22.4% of proteins, respectively, were identified as high abundance CSF proteins and excluded from downstream analyses (Additional files 2 and 3). Among the remaining proteins, 1134 (56.2%) in EV fractions and 702 (55.6%) proteins in corresponding EV-depleted CSF were identified by two or more peptides in at least one subject.

GO analysis and expression annotation of proteins detected with ≥ 2 unique peptide counts in EV fractions and EV-depleted CSF mapped to biological processes including immune responses, inflammation, stress responses, metabolic processes, and mitochondrial functions, as well as cellular components including neurons, myeloid cells, astrocytes, oligodendrocytes, endothelial cell, epithelial cells, and vesicles (Fig. 2b, c). Given that HIV may infect choroid plexus (CP) cells [58, 59] and dysregulate BBB functions [60, 61], CP and BBB markers were manually curated [48, 62–64]. Full lists of proteins in each biological process and cellular component category are shown in Tables 2 and 3 (CSF EV) and Additional files 4 and 5 (EV-depleted CSF). A subset of proteins (25 in EV fractions and 13 in EV-depleted CSF) identified in ≥ 6 subjects by single peptides with > 10% coverage including CD9, CD81, HLA-A, HLA-DRA, DEFA1, INA, MOG, MRC2, REG3A, TIMP1, TIMP2, and OCLN were also included. As expected, more vesicle-related proteins were identified in EV fractions (19%) compared to EV-depleted CSF (9%). Stress and immune responses were the most frequently represented biological processes in both EV (21% of proteins for both) and EV-depleted CSF (27% and 24% of proteins, respectively) (Fig. 2b, c), while epithelial cells and neurons were the most frequently represented cell types in EVs (24% of proteins) and EV-depleted CSF (27% of proteins), respectively.

**Table 2:**
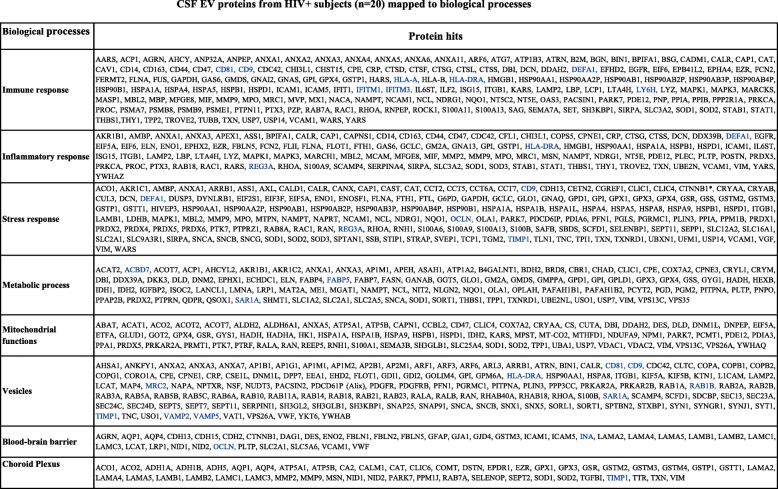
CSF EV proteins from HIV+ subjects (n = 20) mapped to biological processes

Blood-brain barrier and choroid plexus markers were curated manually

Proteins identified by one unique peptide are shown in blue

**Table 3:**
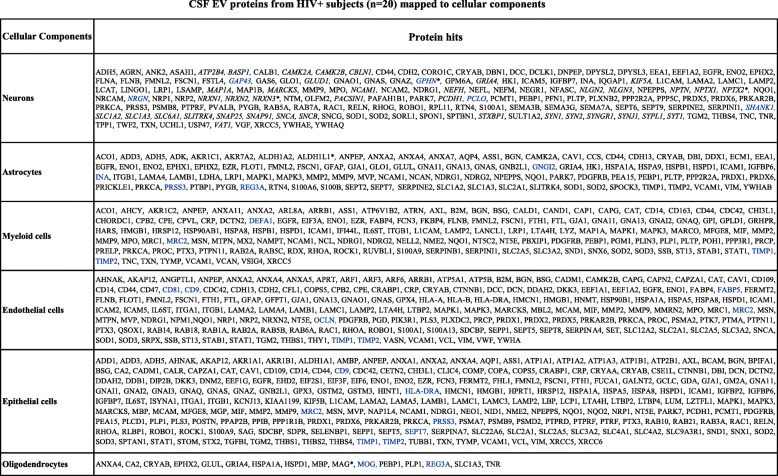
CSF EV proteins from HIV+ subjects (*n* = 20) mapped to cellular components

*Proteins curated manually

Proteins identified by one unique peptide hit are shown in blue

Synaptic proteins are shown in italics

**Fig. 3:**
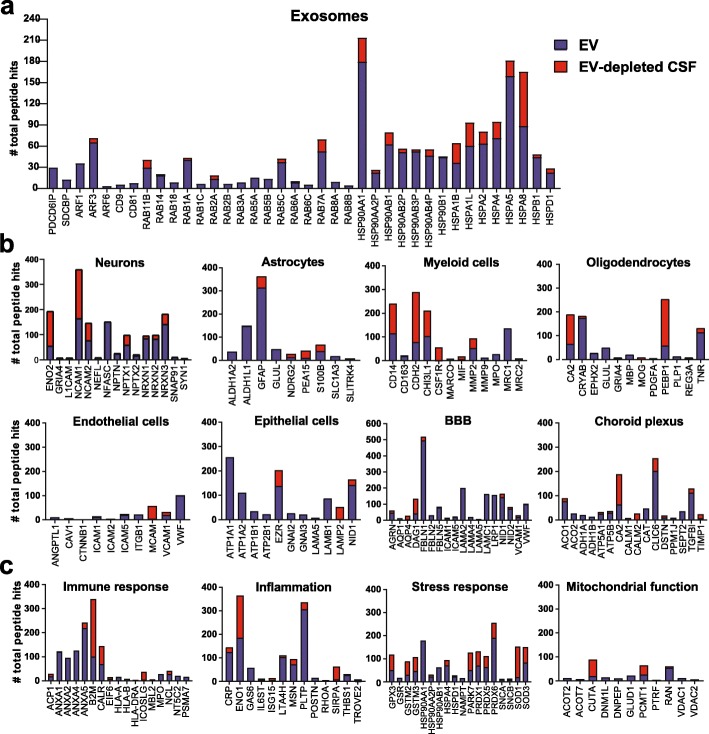
Proteins associated with exosomes, cellular components, and biological processes are abundant in CSF EV fractions. **a** CSF EV fractions are enriched with exosomal proteins compared to EV-depleted CSF. Comparative abundance of proteins related to **b** neurons, astrocytes, myeloid cells, oligodendrocytes, endothelial cells, epithelial cells, blood-brain barrier (BBB), and choroid plexus (CP) and **c** biological processes including immune responses, inflammation, stress responses, and mitochondrial functions in EV fractions and EV-depleted CSF. Bar graphs show the number of total peptide counts for individual proteins among all EV fractions and EV-depleted CSF

Although total peptide counts and percentage of proteins mapped to individual biological process and cellular component categories in EV and EV-depleted CSF were similar, the majority of proteins were more abundant in EV fractions compared to EV-depleted CSF. Proteins related to exosome biogenesis (PDCD6IP, SDCBP, ARF1, ARF3, and ARF6), tetraspanins (CD81 and CD9), RAB proteins, and heat shock proteins were more abundant in EV fractions compared to EV-depleted CSF (Fig. 3a). Several markers of specific cell types, including neurons (L1CAM, NFASC, and synaptic proteins NPTN, NPTXs, NRXNs, SNAP91, SYN1), astrocytes (ALDH1L1, GFAP, GLUL, S100B), myeloid cells (MMP2, MPO, MRC1), and oligodendrocytes (CRYAB, EPHX2, MBP, PLP1, TNR) were identified mainly in EV fractions (Fig. 3b). Endothelial cell (ICAM, VCAM, VWF), epithelial cell (ATP1A, ATP2B, EZR, LAMA, LAMB, LAMP, CLIC6, GNAI), BBB (AGRN, AQP1, AQP4, DAG1, FBLNs, NIDs), and CP (ACO, ATP5, CALM, CAT, CLIC6, SEPT2) markers were also detected in EV fractions, while a subset of cell-type markers including B2M, CD14, CHI3L1, ENO2, NCAM1, NCAM2, NRCAM, PEA15, PEBP1, CA2, and LAMP2 were detected in both EV fractions and EV-depleted CSF. Many proteins related to inflammation (CRP, GAS6, LTA4H, MSN, PLTP, POSTN, TROVE2), immune responses (ACP1, ANXAs, HLAs, MPO, NCI, NTC5C2, PSMA7), stress responses (GSR, HSP70, HSP90, NAMPT, PRDXs, SNCA, SNCB), and mitochondrial functions (ACOT, DNM1L, DNPEP, GLUD1, RAN, VDAC) were more abundant in CSF EVs compared to EV-depleted CSF (Fig. 3c).

### CSF EV protein cargo in relation to HAND

**Fig. 4:**
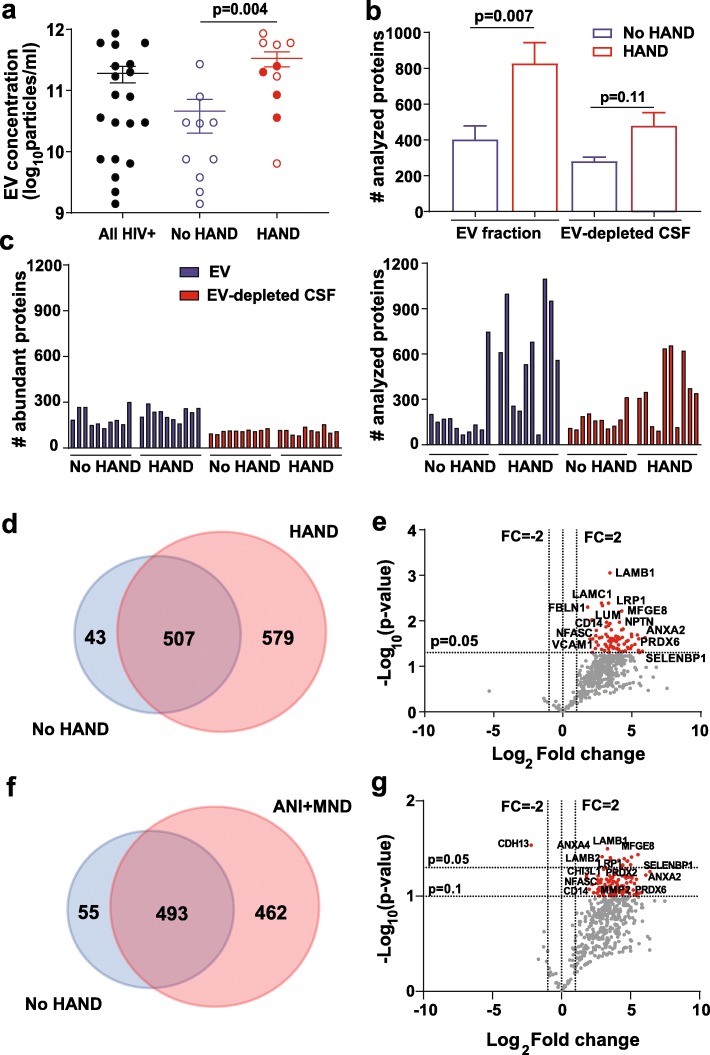
CSF EV protein abundance in relation to cognitive status of HIV+ subjects (*n* = 20). HAND subjects (*n* = 10) had **a** higher EV concentration (solid red circles represent subjects with HAD) and **b** greater EV-associated protein abundance compared to HIV+ subjects without HAND (*n* = 10). **c** The number of high abundance proteins detected in EV fractions and EV-depleted CSF was similar in subjects with versus without HAND (left panel), while HAND subjects had greater abundance of analyzed proteins compared to those without HAND (right panel). **d** Venn-diagram showing overlap of proteins identified in CSF EV fractions from subjects with and without HAND. **e** Volcano plot showing differences in protein abundance for subjects with vs. without HAND among 507 proteins identified in both groups. Each dot represents a single protein; red dots correspond to proteins significantly increased by ≥ 2-fold (*p* < 0.05). Selected proteins with high fold changes, *p* values < 0.05, or biological relevance for HAND pathophysiology are labeled. **f** Venn-diagram showing overlap of proteins identified in CSF EV fractions from subjects with ANI or MND and without HAND. **g** Volcano plot showing differences in protein abundance for subjects with ANI or MND vs. without HAND among 493 proteins identified in both groups. Each dot represents a single protein; red dots correspond to proteins significantly increased by ≥ 2-fold (*p* < 0.1). Selected proteins with high fold changes, *p* values < 0.1, or biological relevance for HAND pathophysiology are labeled

Next, we examined the relationship between the CSF EV proteome and HAND. CSF EV concentrations and number of proteins detected in EV fractions were higher in HIV+ subjects with HAND compared to without HAND (*p* = 0.004 and *p* = 0.007, respectively; Fig. 4a, b). In a sensitivity analysis excluding HAD subjects, CSF EV concentrations remained higher in subjects with milder forms of HAND (ANI + MND) compared to without HAND (Additional file 6: Figure S3a). Comparative size distribution histograms of CSF EVs of representative subjects from each HAND subgroup (Additional file 6: Figure S3b) provided further evidence that subjects with ANI, MND, or HAD had higher CSF EV concentrations compared to those without HAND. Although the number of abundant proteins was similar in subjects with and without HAND (range 160–292 and 129–301, respectively) (Fig. 4c, left), the number of proteins not classified as abundant was greater in HAND subjects (range 67–1098) (Fig. 4c, right). Furthermore, in EV fractions, 579 proteins were exclusively detected in subjects with versus without HAND, compared with 43 proteins detected uniquely in subjects without HAND (Fig. 4d). Among 507 proteins detected in both groups, 491 proteins were increased (77 proteins with ≥ 2 fold change and *p* < 0.05), while 16 were decreased in subjects with HAND compared to those without HAND (Fig. 4e). To evaluate CSF EV profiles in subjects with milder forms of HAND, we reanalyzed the data excluding subjects with HAD. Among 1010 proteins identified by ≥ 2 unique peptides in subjects with mild HAND (ANI + MND) and without HAND, 462 proteins were exclusively detected in subjects with ANI or MND compared to 55 proteins in subjects without HAND. Among 493 proteins detected in ANI + MND as well as non-HAND groups (Fig. 4f), 136 were increased (≥ 2 fold change and low stringency *p* value < 0.1) of which 20 were significantly altered (≥ 2 fold change and *p* < 0.05) in subjects with ANI or MND compared to those without HAND (Fig. 4g). Volcano plots with total number of proteins detected in all subjects with and without HAND (*n* = 1134) or in ANI + MND and no HAND (*n* = 1010) with ≥ 2 peptides are shown in Additional file 6: Figure S3c. While some proteins including ALDH1A1, ANXAs, CD14, LAMB1, LRP1, MAPK1, MFGE8, NFASC, NPTN, PDCD6IP, PRDXs, SELENBP1, and UBE2N were significantly increased or showed similar trends in subjects with HAND (ANI + MND + HAD) or ANI + MND compared to controls, a subset of proteins including CAPN1, CRMP1, LAMB2, RAB10, and CD163 were significantly increased only in the comparison of ANI + MND vs. controls.

**Fig. 5:**
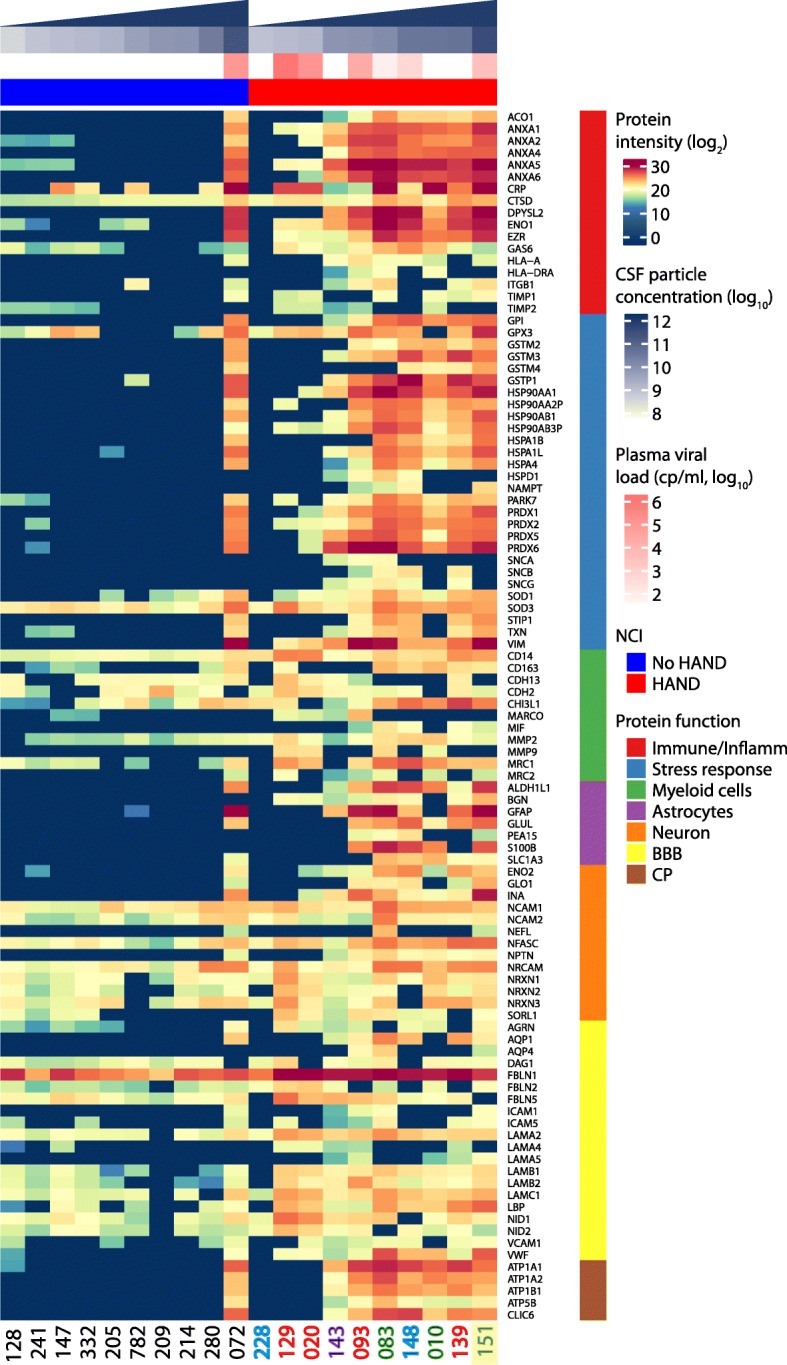
Supervised heatmap of 101 CSF EV proteins identified in 20 HIV+ subjects with (*n* = 10) and without (*n* = 10) HAND. Proteins identified by ≥ 2 unique peptide counts in 6 or more subjects mapping to immune/inflammatory responses, stress response, myeloid cells, astrocytes, neurons, blood-brain barrier (BBB), and choroid plexus (CP) ontology terms are shown. Columns correspond to individual subjects (font color in black: no HAND; green: ANI; blue: MND; red: HAD; purple: NPI-O) and rows to individual proteins. Color scale (blue-yellow-red) illustrates relative log_2_ transformed peptide intensities. CSF EV concentrations (particles/ml) and plasma VL were log_10_ transformed. One HAND subject (ID: 129) had high CSF VL (328 copies/ml) and one non-HAND subject (ID: 072) had high plasma VL (70,953 copies/ml). Triangles at the top illustrate increasing gradient of CSF particle concentrations in subjects with and without HAND. The subject with HIV encephalitis (ID: 151) is highlighted yellow. NCI, neurocognitive impairment

To characterize heterogeneity of proteins mapped to selected cellular components (myeloid cells, astrocytes, neurons, BBB, CP) and biological processes (immune/inflammatory and stress responses) between and within individual HIV+ subjects, we generated supervised heatmaps using the sum of peptide intensities from 101 representative proteins (Fig. 5). Proteins included in this analysis had ≥ 2 peptide counts in CSF EV fractions from 6 or more HIV+ subjects. Eight proteins detected with ≥ 2 peptide counts in < 6 subjects (e.g., MIF, MMP9, NEFL, PEA15, S100B, AQP4, SNCA, SNCB) were also included due to their known importance as markers for respective cell types or biological processes. Most EV proteins including inflammatory/immune response (ANXA, CRP, DPYSL2, ENO1, EZR, HLA, ITGB1, TIMP1), stress response (PARK7, PRDX2, SNCA, SNCB, SNCG, VIM), neuronal (NFASC, NPTN, NRXNs), astrocyte, (ALDH1L1, GFAP, GLUL, PEA15, S100B, SLC1A3) and CP (ATP1A1, ATP1A2, ATP1B1, ATP5B, CLIC6) markers were more abundant in subjects with versus without HAND, a finding that correlated with higher CSF EV concentrations and plasma VL in HAND. However, a subset of neuronal (NCAMs, NRCAM), myeloid cell (CD14, CDH13, CDH2, CHI3L1), and BBB (FBLNs, LAMAs, LAMBs, LAMC1, NIDs) markers were detected in both HAND and non-HAND subjects, largely irrespective of EV concentrations and plasma VL. Unsupervised clustering of these same proteins segregated 9 of 10 HAND subjects (Additional file 6: Figure S4), while one non-HAND subject with high plasma VL and EV concentration clustered with HAND subjects. Several astrocyte markers (ALDH1L1, GFAP, GLUL, S100B, SLC1A3) clustered with stress response (HSP, SOD, PRDX, GSTM, GSTP, GPI, STIP) and inflammation (DPYSL2, ANXA, EZR, ENO1) markers, suggesting inter-relationships between astrocyte-derived EVs, stress responses, and inflammation.

### Astrocyte, stress responses, and inflammation markers in CSF and U87 EVs detected by immunoblotting

**Fig. 6:**
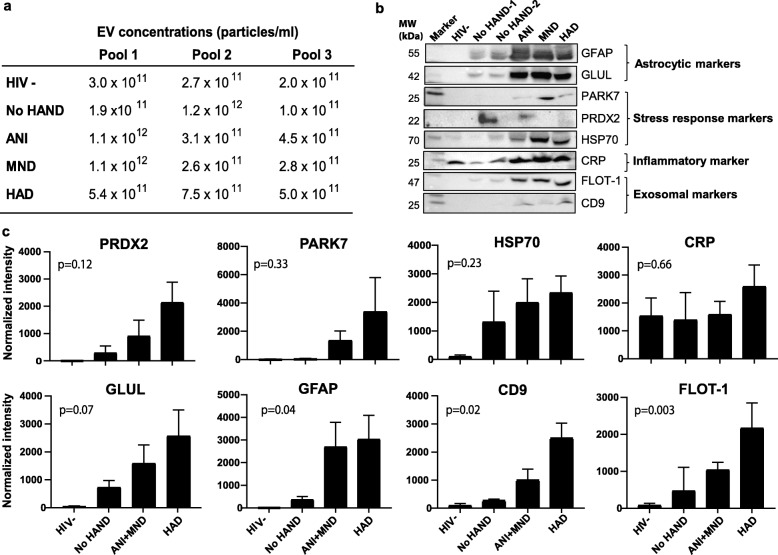
Abundance of astrocytic, stress responses, and inflammatory markers in CSF EVs of HIV- and HIV+ subjects with or without HAND. **a** EV concentrations measured in 300 μl pooled CSF samples by NTA in HIV-, HIV+ non-HAND, and HAND (ANI, MND, and HAD) subjects (*n* = 3; 2–3 subjects per pool). **b** Detection of astrocytic markers GLUL and GFAP, stress response markers PRDX2, PARK7, and HSP70, inflammatory marker CRP, and exosomal markers CD9 and FLOT-1 in CSF EV fractions by immunoblotting. One representative blot is shown for individual markers. Results are representative of three independent experiments. **c** Densitometric quantification of HSP70, PARK7, PRDX2, CRP, GLUL, GFAP, CD9, and FLOT-1 in samples from HIV-, HIV+ non-HAND, ANI + MND, or HAD from immunoblotting (*n* = 3 independent immunoblots, except PARK7, PRDX2, and CD9 where *n* = 2). Bands in each lane were normalized to corresponding EV concentrations. Bars denote mean, error bars denote standard error (SEM). Significant differences between at least two of the four groups are indicated using one-way ANOVA (*p* value < 0.05)

To further investigate the role of astrocyte-derived EVs in stress responses and inflammation in HIV+ subjects with and without HAND, we selected a subset of markers for astrocytes (GFAP, GLUL), stress responses (PARK7, PRDX2, HSP70), and inflammation (CRP) for validation by immunoblotting. For this purpose, we used independent pools of 300 μl CSF from HIV-negative, HIV+ without HAND, and HIV+ with HAND (ANI, MND, and HAD) subjects. CSF EVs from HIV-negative subjects were included as an additional control. Mean EV concentrations from three independent CSF pools are shown in Fig. 6a. Exosome markers CD9 and FLOT-1 were included to detect presence of exosomes in the EV preparations (Fig. 6b). Bands in each lane were normalized to corresponding EV concentrations measured by NTA. Higher levels of CD9 and FLOT-1 were detected in HAND compared to non-HAND and HIV-negative subjects, suggesting higher abundance of exosomes. Increasing trends of PRDX2, PARK7, and HSP70 were detected in CSF EVs of mild (ANI and MND) as well as more severe HAND (HAD) compared to control subjects (Fig. 6b, c). GFAP and GLUL were also detected in CSF EVs, suggesting astrocytes are a potential cellular source of CSF EVs in HIV+ subjects. Furthermore, GFAP and GLUL levels were increased in CSF EVs of subjects with HAND compared to without HAND. These findings suggest that CSF EVs secreted from astrocytes and other cell types in HIV+ subjects carry cargo related to immune/inflammation and stress responses.

**Fig. 7:**
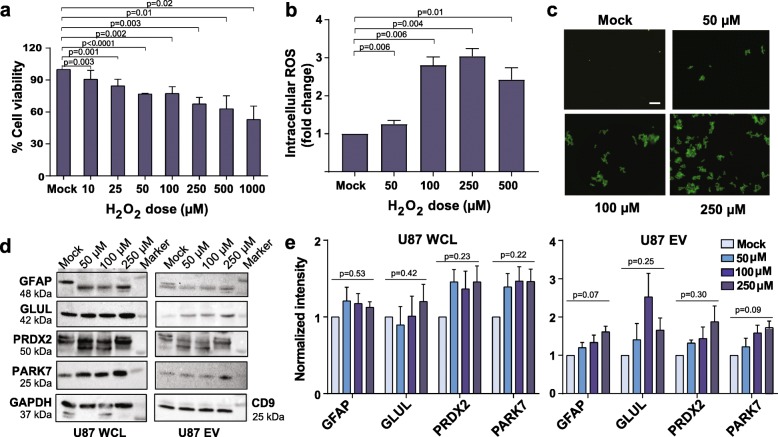
Effect of oxidative stress on astrocytic, stress response, and inflammation markers in U87 cells and EVs. **a** Cultured U87 cells were treated with 10, 25, 50, 100, 250, 500, and 1000 μM H_2_O_2_ for 4 h. Cell viability was measured by MTT assay (*n* = 3). Data represent mean ± SEM. **b** U87 cells were treated with H_2_O_2_ (50, 100, 250, and 500 μM) for 4 h and generation of intracellular ROS was measured spectrophotometrically (*n* = 3). Significant differences in **a** and **b** were evaluated by *t* test (*p* value < 0.05). **c** Fluorescence images of intracellular ROS following H_2_O_2_ treatment (50, 100, 250 μM) of U87 cells for 4 h. Scale bar = 100 μm. **d** Immunoblotting for astrocytic (GFAP, GLUL) and stress response (PRDX2, PARK7) markers in U87 cells and EVs (*n* = 3). GAPDH and CD9 were used as loading controls for U87 cells and U87 EVs, respectively. Results are representative of three independent experiments. **e** Densitometric quantification of normalized GFAP, GLUL, PARK7, and PRDX2 bands from U87 cells and EVs. Significant differences between at least two of the four conditions in each set are indicated using one-way ANOVA (*p* value < 0.05)

To test whether astrocytes are a potential cellular source of EVs carrying inflammation and stress response markers experimentally, mock and H_2_O_2_-treated astrocytic U87 cells and U87-derived EVs were used to further evaluate astrocyte, stress response, and inflammation markers detected in Fig. 6b and c (GFAP, GLUL, PRDX2, PARK7, CRP). U87 cells were treated with 10, 25, 50, 100, 250, 500, or 1000 μM H_2_O_2_ for 4 h, which showed a dose-dependent effect in three independent experiments and induced 3% to 58% cell death with increasing doses (Fig. 7a). Intracellular ROS was detected by H_2_DCFDA staining of U87 cells following treatment with 50, 100, 250, or 500 μM H_2_O_2_ for 4 h. Higher ROS production was observed with increasing H_2_O_2_ concentrations from 50 to 250 μM (Fig. 7b, c). Due to increased cell death, we did not include H_2_O_2_ concentrations higher than 250 μM in further experiments. Control and H_2_O_2_-treated (50, 100, 250 μM) cells and EVs were characterized by immunoblotting for astrocyte (GFAP, GLUL), inflammation (CRP), and stress response (PRDX2, PARK7) markers (Fig. 7d). Bands in each lane were normalized to respective GAPDH bands. Increasing trends in expression of PRDX2 and PARK7 were detected in H_2_O_2_-treated U87 cells and EVs compared to respective controls (Fig. 7e), suggesting that astrocyte-derived EVs can carry these stress response markers under oxidative stress conditions.

## Discussion

In a recent study, we reported that increased CSF EV concentrations correlate with neurocognitive impairment and the neuronal injury biomarker CSF NFL in ART-treated HIV patients [50], suggesting CSF EVs may contribute to HAND pathophysiology. Here, we characterized the CSF EV proteome in HIV+ subjects and its relationship to HAND by untargeted LC/MS/MS analysis. Compared to prior CSF proteomics studies of HIV+ subjects in which < 1000 proteins were detected [16, 18, 19], our proteomics analysis identified > 2700 proteins in CSF EVs, including markers of neurons, astrocytes, myeloid cells, epithelial cells, endothelial cells, and CP. Furthermore, CSF EVs carried proteins related to exosomes (CD9, CD81, FLOT-1, ALIX), synapses (NPTN, NPTXs, NRXNs), inflammation/immune responses (ANXAs, GAS6, CRP, HLAs), stress responses (HSPs, SODs, PARK7, PRDXs, TXN), BBB (AGRN, AQP4, DAG1, VCAM1), and mitochondrial functions (DNM1L, DNPEP, GLUD1, RAN, VDAC). Many of these proteins have low abundance in CSF, but are enriched in CSF EV fractions. CSF EV concentrations and EV-associated proteins linked to these brain cell types and biological processes were increased in HAND compared to non-HAND subjects. These findings suggest that CSF EVs are a valuable source of new biomarkers and may also provide insights into pathological processes involved in HAND.

We detected cell-type markers related to neurons, astrocytes, oligodendrocytes, myeloid cells, CP epithelia, and BBB in CSF EVs from HIV+ individuals, consistent with prior studies demonstrating that EVs originate from various brain cell types and CP [47, 48, 63–66]. The identification of astrocytic (ALDH1A2, ALDH1L1, GFAP, GLUL, EAAT1) and myeloid cell (CD163, MRC1, MIF, MMPs) markers in CSF EVs suggests these cells are potential sources. We also detected neuronal markers and synaptic proteins such as NLGNs, NPTN, NRXNs, NPTXs, and SYN1, which function in synapse formation, plasticity, adhesion, scaffolding, and directional signaling [67–70], as well as markers for choroid plexus (ACO2, ATP, CLIC6, EZR, TTR), epithelial cells (ATP1A1, ATP1A2, GNA12, LAMA2, LAMA5, LAMB1), and endothelial cells (CAV1, ICAM1, VCAM1, VWF) in CSF EVs, indicating these cell types are likely to be additional cellular sources [48, 71]. The average diameter and peak size range of particles in CSF EVs corresponded to the size of exosomes, consistent with our finding that proteins related to exosome biogenesis and release such as Alix (PDCD6IP), syntenin (SDCBP), tetraspanins, ADP-ribosylation factors (ARF), and Rab proteins were enriched in CSF EVs compared to EV-depleted CSF. However, substantial concentrations of particles with smaller (< 50 nm) and larger (> 150 nm) diameters were also detected in CSF EVs, indicating these preparations consist of heterogeneous populations of extracellular vesicles and microparticles.

EVs have emerged as an important source of biomarkers in neurological diseases including Alzheimer’s disease, Parkinson’s disease, MS, amyotrophic lateral sclerosis, and Huntington’s disease [15, 24, 25, 28–30]. However, the association of CSF EVs with HAND is less clear. We detected increased CSF EV concentrations in subjects with milder (ANI + MND) as well as severe forms of HAND compared to without HAND. Higher abundance of EV-associated proteins related to immune/inflammation and stress responses in HAND may reflect increased CSF EV concentrations as well as pathophysiological processes in the CNS. Proteomics analysis showed higher abundance and clustering of astrocytic (ALDH1L1, GFAP, GLUL, PEA15, S100B), immune/inflammatory (ANXA, CRP, DPYSL2, ENO1, EZR, TIMP), and stress response markers (GST, HSPs, PARK7, PRDX, SNCA, SNCB) in CSF EVs of HAND subjects, raising the possibility that higher abundance of these proteins in CSF EVs in HAND compared to non-HAND subjects may reflect reactive astrocytosis, neuroinflammation, and/or oxidative stress [3, 5, 6, 9, 10, 72–75].

We identified immune/inflammation markers including ANXAs, CRP, IL6ST, IL1RL1, HLAs, MMPs, and TIMPs in CSF EVs from HIV patients, consistent with previous studies [15, 75, 76]. Myeloid cells express higher levels of immune/inflammation markers compared to astrocytes and endothelial cells in the CNS [77, 78], representing one potential source of these proteins in CSF EVs. MMPs, together with inflammatory cytokines, disrupt BBB integrity by degrading extracellular matrix and tight junctions [79, 80], thereby promoting CNS infiltration of leukocytes and inflammation-related molecules. We detected significant amounts of CRP, a protein mainly secreted by the liver in response to IL-6 [81], in CSF EVs from HIV+ individuals, which may reflect redistribution of this protein from blood into the CNS during HIV infection [82]. CRP has known pro-inflammatory effects on myeloid cells and is therefore a candidate systemic factor that may promote inflammation in the CNS of HIV patients. Peripheral inflammatory signals can also be transmitted to the CNS via CP epithelia-derived CSF EVs [63]. Thus, our CSF EV proteomics analysis identifies new potential mechanisms that may promote CNS inflammation in HAND via crosstalk between peripheral and CNS compartments.

Inflammation enhances EV abundance in CSF, which in turn can activate astrocytes and worsen neurological disorders [77]. EVs released from reactive astrocytes contain inflammation- and stress-related molecules [37, 73]. In CSF EVs of HAND subjects, we detected increased inflammation and stress response markers including PRDX2, PARK7, HSP70, and CRP by untargeted proteomics and immunoblotting. Increasing trends of these markers were also detected in subjects with milder forms of HAND (ANI or MND). Higher PRDX2 and PARK7 were also detected in EVs isolated from H_2_O_2_-treated astrocytic U87 cells compared to untreated cells, suggesting astrocytes are a potential source of CSF EVs carrying stress response markers. CSF EVs secreted under oxidative stress from glial cells may exert protective effects on neurons [83–85], which have relatively low antioxidant capacity and depend on astrocytes for defense against oxidative stress. EV-associated HSP proteins [86] and other anti-oxidant proteins such as SODs and PRDXs may increase protection from oxidative stress [84, 87] and increase stress tolerance [83, 84]. Astrocytes can also transfer functional mitochondria to neurons through EVs [88]. Our finding that CSF EVs in HAND subjects carry mitochondrial markers is consistent with a prior study reporting mitochondrial proteins in peripheral blood EVs of human T-lymphotropic virus type 1-infected subjects [89]. Further studies are needed to determine if EV-associated mitochondrial proteins represent elimination of damaged mitochondria or mediate cell-cell communication in stress conditions.

We detected common abundant proteins such as albumin, immunoglobulins, apolipoproteins, complements, collagen, and ribonucleoproteins in CSF EV fractions as well as EV-depleted CSF. Although some of these abundant proteins may have functional roles in HAND pathogenesis, we excluded them from downstream analyses for several reasons. High abundance proteins such as albumin, immunoglobulin, and transferrin constitute > 70% of CSF proteins [90] and are frequently blood-derived, while many brain-specific proteins related to disease pathways are present in CSF at much lower concentrations [91]. Excluding high abundance proteins allowed us to focus the analysis on protein patterns and pathways more likely to represent CNS-derived proteins and candidate biomarkers. Albumin, immunoglobulins, α-2-macroglobulin, apolipoproteins, complements, collagen, and ribonucleoproteins were still abundant in CSF EV fractions of HIV+ subjects even after removal of common abundant proteins by immunoaffinity depletion. Given high abundance in CSF, these proteins are likely to be contaminants that precipitate as protein aggregates during EV isolation rather than bona fide EV proteins. In view of these considerations, we compiled a list of common abundant CSF proteins (Additional file 1) and excluded them from downstream analyses.

We acknowledge some limitations of the study. One limitation relates to purity of isolated CSF EVs. The optimal method for isolating EVs from small volumes of CSF is to precipitate vesicles using an EV-precipitating agent to prevent particle loss. However, this method may also precipitate other non-membranous particles and protein aggregates. NTA does not distinguish these aggregates from membrane-bound EVs, which may result in some false positive data and contamination of soluble CSF proteins in the EV fractions. However, low sample volume limited our ability to use ultracentrifugation methods for EV isolation, detect markers present at low levels, and normalize proteomics data to EV concentrations. Although we purified CSF EVs after depleting immunoglobulins and 12 abundant proteins, some high abundance proteins remained in CSF samples. Due to small sample volume, it was challenging to remove all high abundance proteins from CSF samples and the immunodepletion procedure may have reduced EV yields. Further studies with larger sample volumes and sample sizes are needed to overcome these limitations. The study cohort consisted mainly of HIV+ subjects with advanced disease and low nadir and current CD4 counts, long duration of HIV infection, and exposure to older ART regimens, so it is possible that some differences we detected between HAND versus non-HAND subjects are related to the higher prevalence of viremia, “legacy pathologies,” or other factors rather than cognitive status. Additionally, peripheral viremia could have confounding effects on the CSF EV proteome. Further studies of suppressed cohorts on modern ART regimens in which mild forms of HAND predominate are warranted to further define the CSF EV proteome and pathobiology of ANI and MND in contemporary settings.

## Conclusion

This study characterizes the CSF EV proteome in HIV+ subjects with and without HAND. Our findings suggest that CSF EVs in HIV+ individuals are likely to originate from neurons, glial cells, choroid plexus epithelial cells, and BBB and may participate in diverse types of cell-to-cell communication in the CNS. Higher abundance of proteins related to synaptic function, immune/inflammation and stress responses, mitochondria, and BBB in CSF EVs of HAND compared to non-HAND subjects suggests that CSF EVs are likely to be involved in HIV-associated neurocognitive impairment and represent a valuable source of candidate biomarkers for future studies. Although we did not identify HAND-specific biomarkers, our untargeted approach identified a number of interesting CSF EV proteins that warrant further study in large prospective cohorts using sensitive targeted quantitative assays to evaluate their potential to serve as predictive disease-specific markers.

## Supplementary information

**Additional file 1: Table S1.** List of abundant proteins.

**Additional file 2: Table S2.** Mass spectrometry identification of proteins in 20 HIV+ CSF EV fractions using ABSciex 4800Plus MALDI-TOF/TOF platform.

**Additional file 3: Table S3.** Mass spectrometry identification of proteins in 20 HIV+ EV-depleted CSF using ABSciex 4800Plus MALDI-TOF/TOF platform.

**Additional file 4: Table S4.** EV-depleted CSF proteins from HIV+ subjects (*n*=20) mapped to biological functions.

**Additional file 5: Table S5.** EV-depleted CSF proteins from HIV+ subjects (*n*=20) mapped to cellular components.

**Additional file 6: Figure S1.** Depletion of abundant proteins from CSF. **Figrue S2.** Immunoblotting for ER membrane markers calnexin and Erp72, and exosome markers CD81 and CD9 in CSF EVs from 2 representative HIV+ subjects. **Figure S3.** Comparison of CSF EV concentrations and protein abundance in HIV+ non-HAND, ANI + MND, and HAD subjects. **Figure S4.** Unsupervised heatmap of 101 CSF EV proteins identified in 20 HIV+ subjects with (*n* = 10) and without (*n* = 10) HAND.

## Data Availability

The dataset and figures supporting the conclusions of this article are included as additional files.

## Abbreviations

ANI: Asymptomatic neurocognitive impairment
ART: Antiretroviral therapy
BBB: Blood-brain barrier
CNS: Central nervous system
CP: Choroid plexus
CSF: Cerebrospinal fluid
DMEM: Dulbecco’s modified Eagle’s medium
EV: Extracellular vesicle
FBS: Fetal bovine serum
GO: Gene ontology
HAD: HIV-associated dementia
HAND: HIV-associated neurocognitive disorders
HIV: Human immunodeficiency virus type 1
IQR: Interquartile range
MND: Mild neurocognitive disorder
NPI-O: Neuropsychological impairment due to other causes
NTA: Nanoparticle tracking analysis
PBS: Phosphate-buffered saline
ROS: Reactive oxygen species
TEM: Transmission electron microscopy
VL: Viral load
WBC: White blood cell
