# Supplementary material for: Proteomic analysis of cerebrospinal fluid extracellular vesicles reveals synaptic injury, inflammation, and stress response markers in HIV patients with cognitive impairment
Source: J Neuroinflammation. 2019 Dec 5;16:254. doi: 10.1186/s12974-019-1617-y (PMC6896665; doi:10.1186/s12974-019-1617-y)
Supplement: Supplementary file 6 — Additional file 6: Figure S1. Depletion of abundant proteins from CSF. Figrue S2. Immunoblotting for ER membrane markers calnexin and Erp72, and exosome markers CD81 and CD9 in CSF EVs from 2 representative HIV+ subjects. Figure S3. Comparison of CSF EV concentrations and protein abundance in HIV+ non-HAND, ANI + MND, and HAD subjects. Figure S4. Unsupervised heatmap of 101 CSF EV proteins identified in 20 HIV+ subjects with (n = 10) and without (n = 10) HAND. [file 12974_2019_1617_MOESM6_ESM.docx]

**SUPPLEMENTARY MATERIAL**

***Journal of Neuroinflammation***

**Proteomic Analysis of Cerebrospinal Fluid Extracellular Vesicles Reveals Synaptic Injury, Inflammation, and Stress Response Markers in HIV Patients with Cognitive Impairment**

**Debjani Guha^1^, David Lorenz^1^, Vikas Misra^1^, Sukrutha Chettimada^1^, Susan Morgello^2^, and Dana Gabuzda^1.3^**

^1^Department of Cancer Immunology and Virology, Dana-Farber Cancer Institute, Boston, MA

^2^Department of Neurology, Neuroscience and Pathology, Mount Sinai Medical Center, New York, NY

^3^Department of Neurology, Harvard Medical School, Boston, MA

**Correspondence:** Dr. Dana Gabuzda, CLS 1010, 450 Brookline Ave, Boston, MA 02215

Email: dana_gabuzda@dfci.harvard.edu, Tel: 617-632-2154

**Supplementary Figure 1**


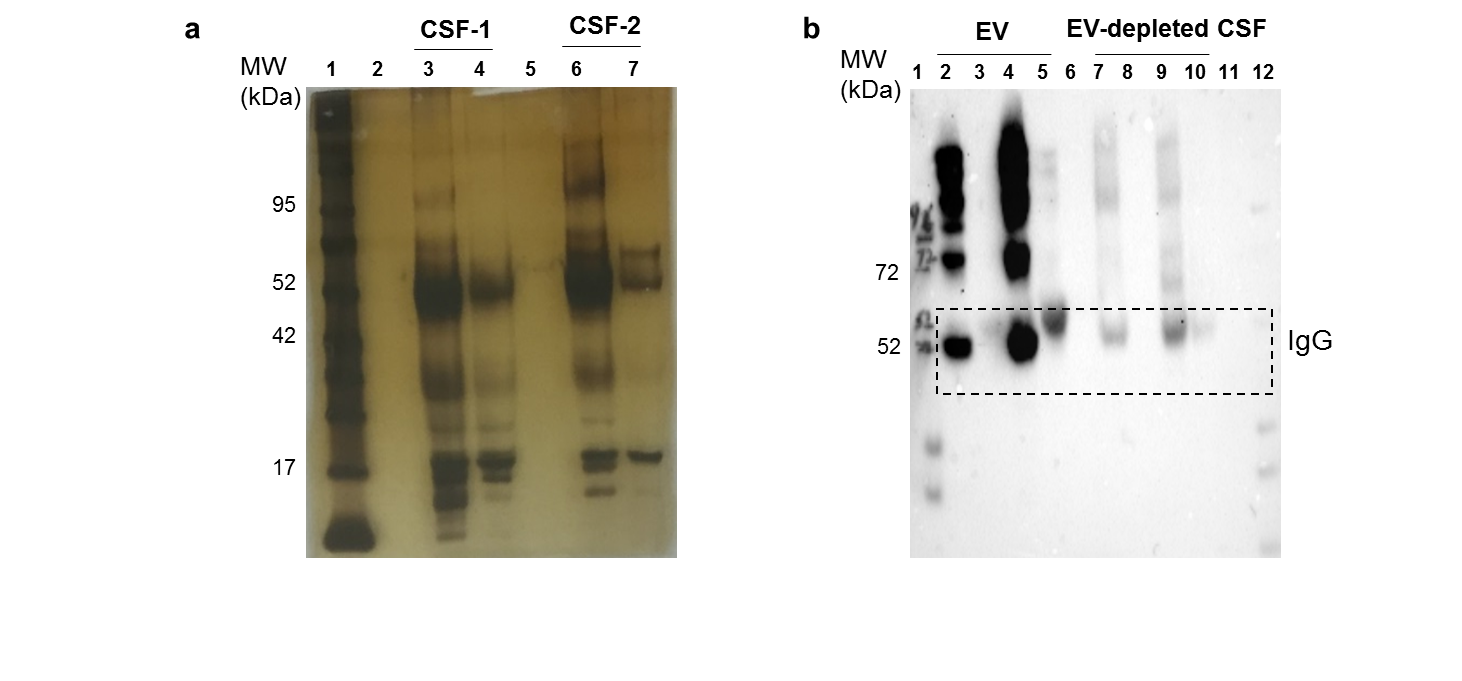


**Fig. S1** Depletion of abundant proteins from CSF. (a) Representative silver stained gel showing protein patterns in CSF of 2 representative HIV+ subjects before and after abundant protein depletion. Lane 3, 6 and 4, 7 show CSF proteins before and after abundant protein depletion, respectively. Lanes 2 and 5 are empty; lane 1 is MW markers (b) Proteins in EV fractions isolated from CSF before and after abundant protein depletion and corresponding EV-depleted CSF samples were separated by SDS-PAGE and immunoblotted for human IgG. Lane 2, 4 show CSF EVs and 7, 9 EV-depleted CSF before abundant protein depletion. Lanes 3, 5 show CSF EVs; lanes 8, 10 EV-depleted CSF after abundant protein depletion; lane 11 is empty; lanes 1, 12 are MW markers.

**Supplementary Figure 2**

**
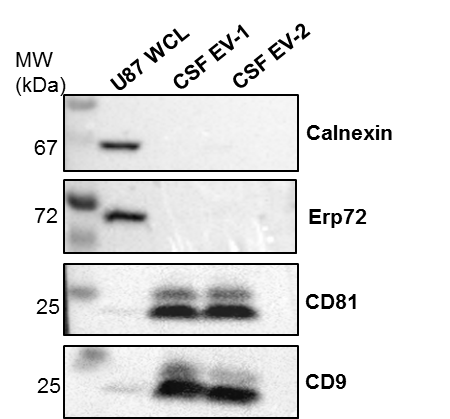
**

**Fig. S2** Immunoblotting for ER membrane markers calnexin and Erp72, and exosome markers CD81 and CD9 in CSF EVs from 2 representative HIV+ subjects. U87 cell lysate was used as a positive control. Calnexin and Erp72 were not detected in CSF EV fractions.

**Supplementary Figure 3**


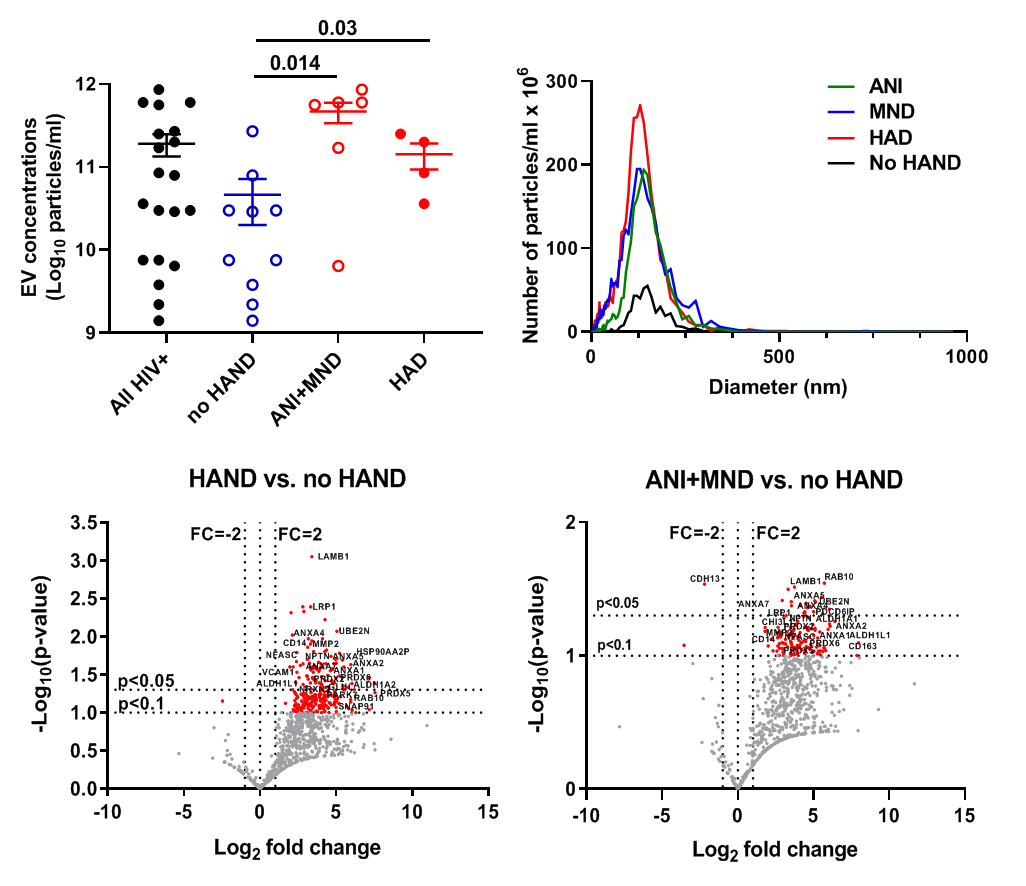


**Fig. S3** Comparison of CSF EV concentrations and protein abundance in HIV+ non-HAND, ANI+MND, and HAD subjects. (a) CSF EV concentration in relation to cognitive status of HIV+ subjects (n=20). Subjects with HAD (n=4) and ANI+MND (n=6) had higher EV concentrations compared to subjects without HAND (n=10). (b) Histograms of CSF EV size distribution of representative subjects from each HAND subgroup. (c) Volcano plots showing differences in protein abundance among 1134 proteins for subjects with vs. without HAND (ANI+MND+HAD; left panel) or 1010 proteins for subjects with ANI or MND vs. without HAND (right panel). Each dot represents a single protein; red dots correspond to proteins increased by ≥ 2-fold (p<0.1). Selected proteins of interest with high fold-changes (>10 fold), p-values <0.1, or possible biological relevance for HAND are labeled.


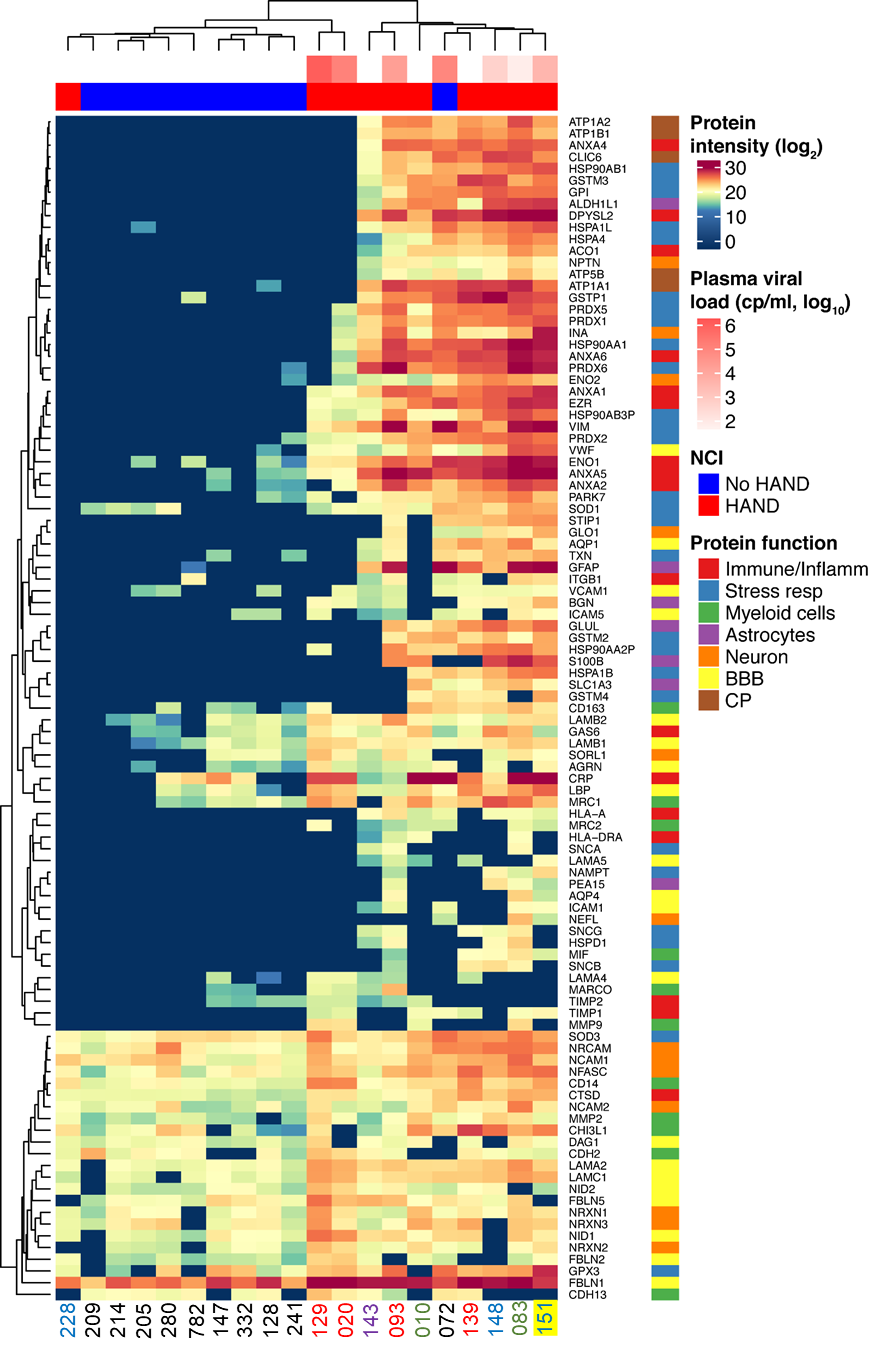


**Supplementary Figure 4**

**Fig. S4** Unsupervised heatmap of 101 CSF EV proteins identified in 20 HIV+ subjects with (n=10) and without (n=10) HAND. Proteins identified by ≥ 2 peptide counts in 6 or more subjects and related to immune/inflammatory response, stress response, myeloid cells, astrocytes, neurons, blood-brain barrier (BBB), and choroid plexus (CP) are shown. Columns correspond to individual subjects (fonts in black: no HAND; green: ANI; blue: MND; red: HAD; purple: NPI-O), rows to individual proteins. The color scale (blue-yellow-red) illustrates the relative log_2_ transformed peptide intensities. Plasma VL was log_10_ transformed. One HAND subject (ID: 129) had high CSF VL (328 copies/ml) and one non-HAND subject (ID: 072) had high plasma VL (70953 copies/ml). The subject with HIV encephalitis (ID: 151) is highlighted yellow. NCI: neurocognitive impairment.
